# Confirmation of *GLRA3* as a susceptibility locus for albuminuria in Finnish patients with type 1 diabetes

**DOI:** 10.1038/s41598-018-29211-1

**Published:** 2018-08-17

**Authors:** Niina Sandholm, Jani K Haukka, Iiro Toppila, Erkka Valo, Valma Harjutsalo, Carol Forsblom, Per-Henrik Groop

**Affiliations:** 1 Folkhälsan Institute of Genetics, Folkhälsan Research Center, FI-00290 Helsinki, Finland; 2 Abdominal Center, Nephrology, University of Helsinki and Helsinki University Hospital, FI-00290 Helsinki, Finland; 3 Research Programs Unit, Diabetes and Obesity, University of Helsinki, FI-00290 Helsinki, Finland; 4 The Chronic Disease Prevention Unit, National Institute for Health and Welfare, FI-00271 Helsinki, Finland; 5 Department of Diabetes, Central Clinical School, Monash University, Melbourne, Victoria Australia

## Abstract

Urinary albumin excretion is an early sign of diabetic kidney disease, affecting every third individual with diabetes. Despite substantial estimated heritability, only variants in the *GLRA3* gene have been genome-wide significantly associated (*p*-value < 5 × 10^−8^) with diabetic albuminuria, in Finnish individuals with type 1 diabetes; However, replication attempt in non-Finnish Europeans with type 1 diabetes showed nominally significant association in the opposite direction, suggesting a population-specific effect, but simultaneously leaving the finding controversial. In this study, the association between the common rs10011025 variant in the *GLRA3* locus, and albuminuria, was confirmed in 1259 independent Finnish individuals with type 1 diabetes (*p* = 0.0013), and meta-analysis of all Finnish individuals yielded a genome-wide significant association. The association was particularly pronounced in subjects not reaching the treatment target for blood glucose levels (HbA_1c_ > 7%; N = 2560, *p* = 1.7 × 10^−9^). Even though further studies are needed to pinpoint the causal variants, dissecting the association at the *GLRA3* locus may uncover novel molecular mechanisms for diabetic albuminuria irrespective of population background.

## Introduction

Diabetic nephropathy (DN) is a devastating disease affecting one third of the individuals with diabetes, with up to 20% of subjects with type 1 diabetes developing end-stage renal disease (ESRD) requiring dialysis treatment or renal transplantation for survival^1^. DN is classically characterized by onset of albuminuria, and subsequent loss of glomerular filtration rate (GFR). Even though loss of renal function may occur also without albuminuria, albuminuria remains an important early predictor of decline of renal function in diabetes^2^. Importantly, elevated albuminuria levels, even within the normal range (albumin excretion rate [AER] < 30 mg/24 hours), predict higher renal risk^3^. Furthermore, already moderately increased AER, i.e. microalbuminuria (30–300 mg/24 hours), is associated with 3-fold increased mortality rate compared with diabetic individuals with normal AER, and patients with macroalbuminuria (AER > 300 mg/24 hours) or ESRD have as high as six to fourteen-fold mortality risk, respectively^4^. There is also an increasing body of evidence that albuminuria may play a pathogenic role in renal disease^2^. Thus, it is of importance to target treatment already at the early stage of DN. However, the treatment options for DN are currently mainly limited to antihypertensive medication, and the development of novel medications has proved challenging as the pathology of DN remains poorly understood.

Albuminuria has been shown to have a genetic component, with heritability estimates of 30% to 45% in individuals with type 2 diabetes^5,6^, and 27% in type 1 diabetes^7^. However, genome-wide association studies (GWAS) have identified only a few genetic susceptibility loci for DN with genome-wide statistical significance, i.e. *p*-value < 5 × 10^−8^, with the main findings often related to the most extreme ESRD phenotype^7–10^. However, a recent GWAS on albuminuria as continuous trait found suggestive evidence of association between albuminuria and variants in the *RAB38* and *HS6ST1* genes (*p*-values < 10^−6^) in the subset of individuals with diabetes^11^.

Our previous GWAS on albuminuria, as measured by 24-hour AER, identified five single nucleotide polymorphisms (SNPs) in the *GLRA3* gene that were associated with albuminuria with genome-wide significance (*p*-value = 1.5 × 10^−9^ for rs10011025) in Finnish individuals with type 1 diabetes in the Finnish Diabetic Nephropathy Study (FinnDiane). Replication in 598 additional FinnDiane individuals with AER measured from timed overnight urine collections (nu-AER) showed a non-significant trend but indeed in the same direction. On the contrary, a replication attempt in 3152 non-Finnish European individuals with type 1 diabetes reached a nominally-significant *p*-value of 0.03 for a directly genotyped SNP, rs1564939 in the *GLRA3* gene, but with the opposite allele associated with albuminuria than in the Finnish subjects. Therefore, the association could not be considered replicated, and remained inconclusive despite the originally genome-wide significant results^7^.

We hypothesized that the observed associations with the common SNPs on the *GLRA3* gene in fact reflect population specific effects and may therefore vary on effect direction. In order to study this topic further, we here assessed the associations of the previously identified genetic variants in additional Finnish individuals recently recruited into the FinnDiane study in order to confirm or to refute the role of these variants for AER in Finnish patients with type 1 diabetes. We further dissected the flanking region to detect low frequency and rare variants contributing to the association seen at the common rs10011025 variant.

## Results

### Replication of the previous results

We previously selected for replication three SNPs genome-wide significantly associated with albuminuria (*p* < 5 × 10^−8^), plus rs11725853 (*p* = 1.8 × 10^−7^), from the *GLRA3* locus. All were in notable linkage disequilibrium (LD) with the lead SNP rs10011025 in the 1000 Genomes Finnish individuals (r2 = 0.78–0.94, D′ = 0.90–1), while in moderate to high LD in British individuals (r2 = 0.61–0.97, D′ = 0.93–1)^12^. In this study, we identified 902 newly recruited FinnDiane patients with 24-hour AER measurements, and 357 patients with overnight nu-AER measurements, and with GWAS data available (Table 1). It is of note that these patients had not been included in the previous GWAS or in the previous replication analysis. In a meta-analysis of these two novel replication sets, all four abovementioned SNPs from the *GLRA3* region were associated with AER (*p* < 0.004) with the direction of effect in line with the original GWAS analyses, i.e. the minor alleles were associated with higher levels of AER (Table 2). As in the original GWAS analysis, the strongest association was obtained for rs10011025 with each additional minor G allele estimated to increase log_10_(AER) by 0.12 (95% confidence interval [CI] 0.05–0.19), equal to multiplying raw AER by 1.12.

**Table 1:** Characteristics of the novel replication patients.

Characteristic	24-hour AER	nu-AER	P
N	902	357	
Men (%)	457 (50.7)	176 (49.3)	NS
Age at onset of diabetes (years)	16.7 ± 9.5	14.9 ± 8.9	0.001
Age (years)	38.2 ± 12.3	42.1 ± 12.6	<0.001
Duration of diabetes (years)	21.5 ± 11.0	27.2 ± 12.1	<0.001
AHT medication (%)	288 (31.9)	183 (51.3)	<0.001
SBP (mmHg)	132.6 ± 17.3	136.1 ± 17.8	0.0025
DBP (mmHg)	78.9 ± 9.6	78.0 ± 10.4	NS
HbA_1c_ (%)	8.5 ± 1.4	8.5 ± 1.4	NS
HbA_1c_ (mmol/mol)	69.3 ± 15.0	69.1 ± 15.2	NS
Mean HbA1_c_ (%)	8.5 ± 1.3	8.5 ± 1.2	NS
Mean HbA1_c_ (mmol/mol)	69.6 ± 13.9	69.3 ± 13.6	NS
Number of HbA1_c_ measurements	8 (2, 18)	15 (6, 31)	<0.001
Retinal laser treatment (%)	20.8%	35.2%	<0.001
24 h AER (mg/24 h), mean ± SD	119 ± 523		
24 h AER (mg/24 h), median (IQR)	9 (5, 26)		
nu-AER (μg/min), mean ± SD		114 ± 698	
nu-AER (μg/min), median (IQR)		8 (3, 24)	

Characteristics were collected at the same visit as the 24-hour/nu-AER. Mean HbA_1c_ refers to mean of all available HbA_1c_ since the onset of diabetes. Data are given as numbers (percent), or mean ± standard deviation (SD), or medians (interquartile range [IQR]). AHT: anti-hypertensive. SBP: systolic blood pressure; DBP: diastolic blood pressure. P: difference between the replication sets; p-values > 0.05 are indicated as non-significant (NS); calculated with Welch two sample t-test for continuous variables, and Pearson’s Chi-squared test for binary variables.

**Table 2:** Replication and meta-analysis results for the SNPs in the GLRA3 locus selected for replication in the original study.

SNP	REF	EA	r^2^	1) Meta novel replication		2) Meta all Finnish replication		3) Meta all Finnish			
				P	β (95% CI)	P	β (95% CI)	P	β (95% CI)	EAF	P_HET_
rs10011025	A	G	0.93	0.0013	0.119 (0.047–0.192)	0.0042	0.093 (0.029–0.157)	4.25 × 10^–10^	0.147 (0.101–0.193)	0.16	0.024
rs11725853	G	A	0.99	0.0032	0.100 (0.034–0.167)	0.0053	0.081 (0.024–0.137)	1.08 × 10^−8^	0.113 (0.074–0.151)	0.20	0.179
rs12509729	G	A	0.92	0.0032	0.107 (0.036–0.178)	0.0103	0.085 (0.020–0.149)	3.66 × 10^−8^	0.139 (0.090–0.189)	0.16	0.025
rs1564939	T	C	0.97	0.0033	0.105 (0.035–0.175)	0.0073	0.082 (0.022–0.142)	1.44 × 10^−9^	0.126 (0.085–0.166)	0.18	0.032

Results are given for (1) meta-analysis of the two novel replication cohorts (24-hour AER and nu-AER, N = 902 and 357, respectively); (2) meta-analysis of these and the previous replication (nu-AER, N = 598; ref.^7^); and (3) meta-analysis of all Finnish cohorts, including the original GWAS finding (24-hour AER, N = 1925, ref.^7^), previous replication study, and the two current replication sets.

REF: Reference allele; EA: Effect allele; r^2^: imputation quality estimate. β: effect size estimate. Positive β indicates that effect allele (EA) is associated with higher AER. β is calculated for log10 transformed AER values, such that β = 0.119 indicates 1.119 fold change in AER per each additional copy of EA. 95% CI: 95% confidence interval. EAF: Mean effect allele frequency in meta-analysis. P_HET_: p-value for heterogeneity.

### Meta-analysis with the previous results

**Figure 1:**
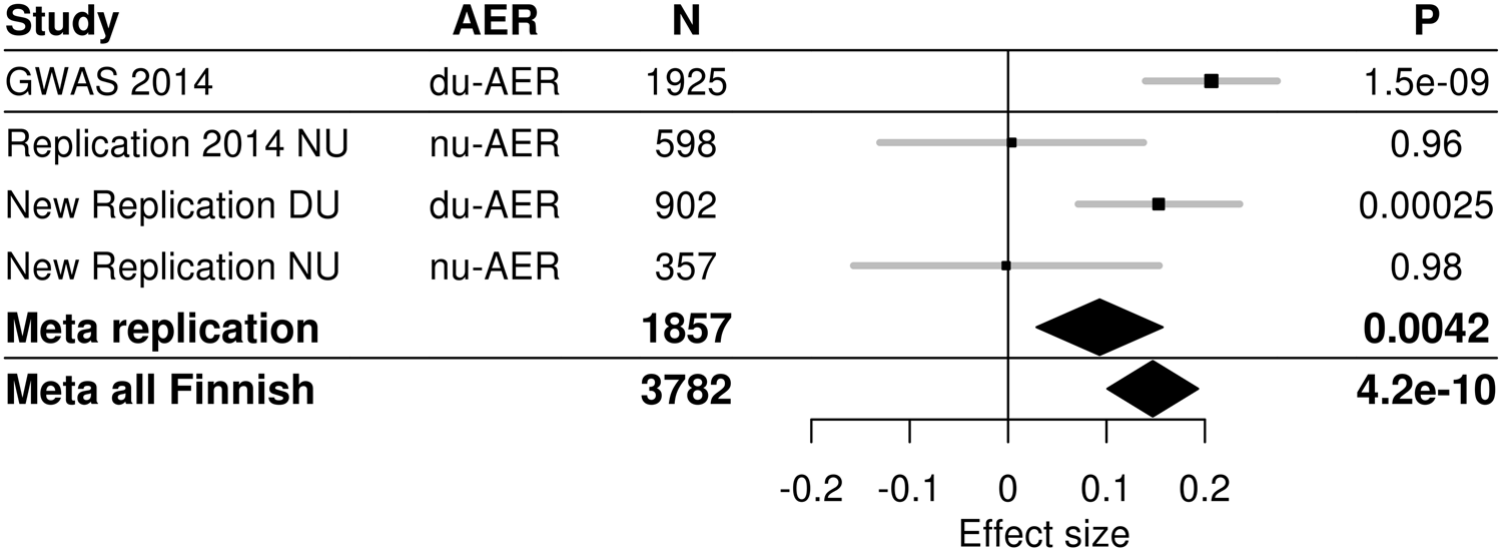
Forest plot of association between rs10011025 and AER in the original discovery cohort (GWAS 2014), in the previously reported Finnish replication set (“Replication 2014 NU”), and in the two novel Finnish replication sets (“New replication DU”, and “New replication NU”). DU: 24-hour urine. Meta replication: meta-analysis of all three replication cohorts; Meta all Finnish: meta-analysis of original discovery study and the three Finnish replication sets.

Meta-analysis with the previous replication results in the 598 Finnish patients were significant (*p* < 0.05) for all four SNPs, with *p* = 0.0042 for rs10011025. Finally, meta-analysis of all Finnish patients improved the original GWAS results yielding a p = 4.25 × 10^−10^ for rs10011025 along with genome-wide significant *p*-values also for the three other SNPs (Table 2). Modest heterogeneity was observed in the meta-analysis (*p*-value for heterogeneity 0.02 for rs10011025), with the two largest sets of 24-hour AER measurements driving the signal (Fig. 1).

As the previous GWAS was imputed with the hapMap2 CEU population as the reference panel, we reanalysed the *GLRA3* region in our novel GWAS, which was imputed using the denser 1000Genomes as reference panel^12^. Combining all subjects from the discovery and the replication steps, this novel GWAS included a total of 2864 patients with 24 hour AER, and in addition 748 patients with overnight nu-AER available, overlapping with the original report. In the meta-analysis of these two sets, the rs10011025 remained the most strongly associated SNP with a *p*-value of 3.29 × 10^−8^.

When we performed conditional analysis adjusting for rs10011025, 47 SNPs in *GLRA3* remained nominally significantly associated with AER (*p* < 0.05), with the strongest residual association seen at the intronic rs112400253 (*p* = 0.0012, β [95% CI] = −0.277 [−0.445 – −0.109], minor allele frequency [MAF] = 0.024). However, as the association did not remain significant after correction for multiple testing (1192 SNPs tested), there seems to be no major associations on the locus independent of rs10011025.

### Stratification by HbA_1c_

**Figure 2:**
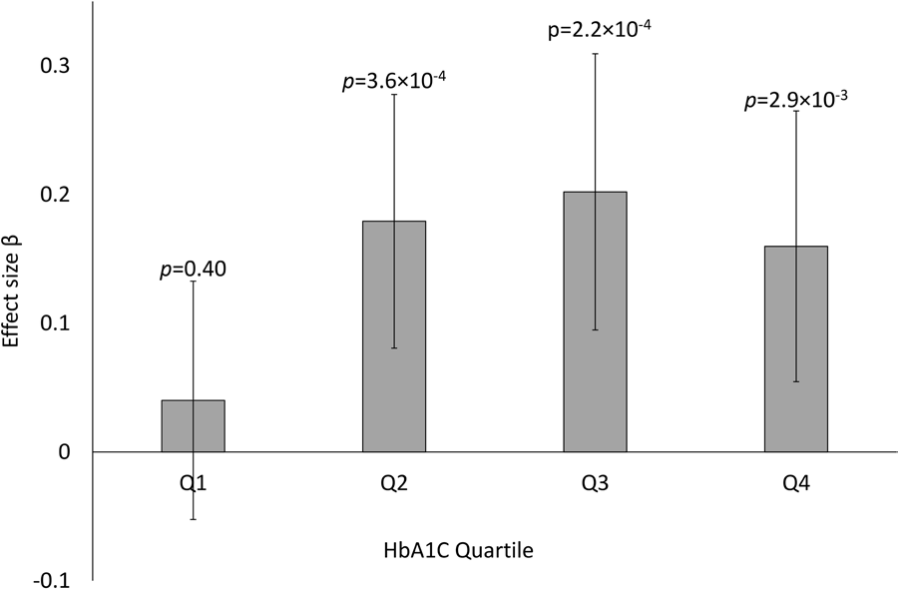
Effect size estimates for association between rs10011025 and 24-hour AER, stratified by the mean HbA1c quartiles. A total of 2864 subjects with updated GWAS data, 24-h AER, and HbA_1c_ were included in the analysis. Q1: 4.92 ≥ HbA_1c_ < 7.68; Q2: 7.68 ≥ HbA_1c_ < 8.42; Q3: 8.42 ≥ HbA_1c_ < 9.32; Q4: 8.42 ≥ HbA_1c_ ≤ 16.30. N = 706 (705) in each quartile.

As the locus showed previously no evidence of association in the non-diabetic individuals, it suggests that the underlying genetic factor only affects albuminuria under diabetic conditions^7,13^. We therefore stratified the analysis further by HbA_1c_ in the combined set of 2864 patients with updated GWAS data and 24-hour AER measurements available. While no association with AER was seen in the patients reaching the current recommended treatment target of an HbA_1c_ ≤ 7.0% (N = 304, β [95% CI] = −0.017 [−0.137–0.101], p = 0.78), the association was highly significant (*p* = 1.7 × 10^−9^) in the patients with an HbA_1c_ > 7.0% (N = 2560, β [95% CI] = 0.17 [0.117–0.230]). The effect size was similar in all the three highest quartiles (7.7% ≤ HbA1c ≤ 16.3%; Fig. 2). Interestingly, association was significant and of similar magnitude across all diabetes duration quartiles, ranging from 10 to 66 years (Supplementary Fig. S1).

### Fine-mapping of the associated region with imputation

One possible explanation for the fact that the association between rs10011025 and AER was only seen in Finnish, and not in non-Finnish Europeans, could be that the variant is tagging population specific low frequency variants. As the causal variants may reside far away from the association signal^14^, we explored a 1 M base pair (bp) region up- and downstream of rs10011025 in the GWAS data. Indeed, there was an enrichment of nominally significant associations across all frequency categories (rare, low frequency, uncommon, common; Supplementary Fig. S2), supporting the possibility that rare/low frequency variants interact with, or underlie the association observed at the common rs10011025. We identified ten missense variants within a 1Mbp region of rs10011025, however, many of these were rare and none was associated with AER (*p* > 0.05; Supplementary Table S1).

### Finemapping of the associated region with whole exome sequencing (WES)

As rare variants are not always well captured by GWAS imputation, we searched for low frequency and rare missense variants in the flanking region in WES data including 328 FinnDiane individuals with data on 24-hour AER, enriched for individuals with DN. As the lead variant rs10011025 is an intronic SNP on the *GLRA3* gene, it was not captured by the WES. Two missense mutations were found in the *GLRA3* gene in the WES data (rs144082170: NM_006529 Val449Ile, MAF = 0.018, minor allele count [MAC] = 12; and rs142149685 NM_006529 Arg368His, MAF = 0.003, MAC = 2). However, neither of them was significantly associated with 24-hour AER (*p*-values 0.46 and 0.81, respectively).

As the statistical power is rather low to detect association at single rare variants, we performed gene aggregate tests to detect burden of missense variants in genes near rs10011025. WES included 7 genes within 1Mbp from the rs10011025; 4 of these genes had missense variants in our WES subjects (*CEP44*, *HPGD*, *ADAM29*, and *GLRA3*). Gene aggregate test with SKAT-O suggested enrichment for missense variants in *ADAM29*, with three missense variants with MAF < 0.10 yielding an aggregate *p*-value of 0.0093 (Supplementary Table 2), significant (*p* < 0.05) after correction for four genes. One of these variants, Leu691 deletion (rs200852076), was nominally associated (*p* = 0.015) with AER also alone. Adjusting the association at *GLRA3* rs10011025 for the Leu691 deletion attenuated the association (rs10011025 β = 0.145, *p* = 0.158 in WES patients; rs10011025 adjusted for Leu691del β = 0.042, *p* = 0.680; Supplementary Fig. S3), suggesting that missense variants in *ADAM29* may contribute to the association observed at the *GLRA3*. As the Leu691 deletion is common in Finnish subjects (MAF = 0.096 in 1000 Genomes^12^, MAF = 0.085 in WES), but has low frequency in non-Finnish Europeans (MAF 0.010–0.028 in 1000 Genomes data^12^), it might also contribute to the potential population differences. However, as the Leu691 deletion is only in partial LD with rs10011025 in *GLRA3* (r^2^ = 0.22, D′ = 0.70 in the 1000 genomes Finnish population^12^), it is unlikely to fully explain the observed association.

## Discussion

We have previously identified genetic variants in the *GLRA3* gene to be genome-wide significantly associated with AER in a Finnish GWAS discovery population (rs10011025 p = 1.5 × 10^−9^), but we were not able to replicate the signal in 598 additional Finnish individuals with type 1 diabetes. On the contrary, the association had a nominally significant association in the opposite direction in non-Finnish Europeans (N = 5077, p = 0.028), leaving this association vague and of uncertain relevance. In the present study including 902 + 357 new Finnish subjects with 24-hour AER and overnight nu-AER measurements available, respectively, we were able to validate the original finding (replication *p* = 0.0013, β [95% CI] = 0.119 [0.047–0.192]), yielding a genome-wide significant association in a meta-analysis of all Finnish patients with type 1 diabetes with GWAS and data on AER available. Furthermore, re-analysis on our updated GWAS platform that now included a total of 3612 individuals with type 1 diabetes and AER measurements available, and by using an improved imputation panel (1000 genomes samples) still suggested rs10011025 to be the lead SNP on the *GLRA3* locus. Conditional analysis did not reveal any other major independent associations on the locus.

The association at rs10011025 with albuminuria seems to be specific to diabetes: No evidence of association was found in non-diabetic patients of European descent^7,13^. Of note, no GWAS on AER has been reported in non-diabetic Finnish subjects. When we evaluated the association stratified by HbA_1c_, the association was particularly strong for patients with HbA_1c_ > 7% (*p* = 1.7 × 10^−9^), whereas no association was seen in those with an HbA_1c_ below the currently recommended treatment target of 7%. A similar difference for p-value and effect size was seen for the lowest HbA_1c_ quartile compared with other equally sized quartiles.

This study included sets of patients with either overnight, or 24-hour urine collection. While the combined effects in both replication and in meta-analysis were statistically significant, the association signal was driven by the 24-hour AER collections (Fig. 1). This may be a question of smaller sample size in the overnight collections; or something specific for the 24-hour collection. Importantly, the mean HbA_1c_ was similar for individuals in the novel 24-hour and overnight AER collections, thus not explaining the difference. It is of note that the majority of the previous non-Finnish replication cohorts were based on overnight urine collections^7^. Interestingly, even light to moderate exercise is known to acutely increase albuminuria due to excess hemodynamic pressure^15^. Thus it is possible that the carriers of the rs10011025 minor variant are more sensitive to hemodynamic pressure and the effect of exercise, a phenomenon not observable in the overnight urine collected during rest.

The different results between the Finnish and the non-Finnish European subjects may also reflect population differences. Due to population isolation and multiple recent bottlenecks, the Finnish population is genetically homogenous and differs from the non-Finnish European population^16,17^. Even though Finns have fewer variant sites in the exomes, the variants that passed the population bottlenecks were enriched in frequency, resulting in a higher number of loss-of-function variants found in an average Finnish individual^17,18^.

While the emerging trans-ethnic GWAS meta-analyses suggest that the majority of GWAS associations show consistent effect across different populations, also population specific associations have been identified^19,20^. For example, in a trans-ethnic GWAS meta-analysis on HbA_1c_, the lead variant in *TMEM79* was genome-wide significantly associated with HbA_1c_ in the East-Asian population, and yielded a significant association also in the trans-ethnic meta-analysis allowing for population differences, but showed an association in the opposite direction in the European population (*p* = 0.0169)^20^. While the nominally significant association in the opposite direction may represent a false positive chance finding (both for our AER finding and for the HbA_1c_ *TMEM79* locus), the potential explanations for population specific effects include synthetic associations, i.e. that the lead locus reflects one or more (population specific) unobserved lower-frequency causal alleles with larger effects; or population specific gene – environment; or gene – gene interactions. Population specific gene – environment interactions are unlikely to explain our rs10011025 (*GLRA3*) finding, as the general environment and treatment can be considered similar in Finland as in other European countries. Gene – gene interactions may explain this, but these are hard to detect, especially if the variant interacting with rs10011025 (*GLRA3*) is a low frequency or rare variant, and not found in other ethnic groups.

To study the impact of rare variants for the association at *GLRA3* rs10011025, we explored the 1Mbp region flanking rs10011025 in 328 FinnDiane patients with WES data. We identified two missense variants in the *GLRA3* gene, but neither of them was significantly associated with AER in this data set; of note, the minor allele counts of these variants were very low, 12 and 2 copies, and thus, we had low power to detect association. There was a significant burden of rare missense variants associated with AER in the *ADAM29* gene (SKAT-O *p* = 0.009), which is located only 185kbp from rs10011025. Further studies are needed to pinpoint the true causal variants behind the GWAS association signal, whether affecting *ADAM29*, *GLRA3*, or another nearby gene.

*ADAM29*, encoding the disintegrin and metalloproteinase domain-containing protein 29, is a transmembrane protein highly expressed in testis. In a genome-wide expression study on kidney diseases^21^ accessed through the NephroSeq data base^22^, *ADAM29* was expressed at low levels both in kidney glomeruli and tubuli; moderate under-expression of *ADAM29* was detected in glomeruli of individuals with other kidney diseases (Focal Segmental Glomerulosclerosis, N = 25, *p* = 1.2 × 10^−4^; lupus nephritis, N = 32, *p* = 1.7 × 10^−4^, gene rank top 8% for both analyses).

The *GLRA3* gene encodes the α3 subunit of glycine receptors (GlyR), which are ligand-gated chloride channels triggered by extracellular glycine, an inhibitory neurotransmitter. In addition to their important role in the central nervous system, the GlyR have also many other functions, as reviewed by Van den Eynden *et al*.^23^. Even though the exact molecular mechanisms remain unclear, ischemia is thought to cause molecular perturbations in the GlyR channels, leading to porous defects in the plasma membranes, and eventually to cell death. Glycine can protect cells from ischemic cell death, and this cytoprotective effect has been reported in renal cells, hepatocytes, and endothelial cells^23^. Furthermore, the cytoprotection was attenuated after inhibition of endogenous GlyR expression by RNA interference in Madin–Darby canine kidney (MDCK) cells^24^. *In vivo* experiments in rats suggest that glycine increases the effective renal plasma flow and GFR, and decreases proximal and distal tubular sodium reabsorption, potentially through an increase in the renal interstitial hydrostatic pressure^25^. This might also link to the fact that the association was driven by individuals with 24-h AER collection, in which albuminuria may be elevated due to increased hemodynamic pressure induced by exercise^15^.

One limitation of the study is the unspecific AER lowering effect of anti-hypertensive medication, which we cannot fully account for. To take this into account, the analyses were adjusted for the use of anti-hypertensive medication; furthermore, when multiple visits were available, we chose the time point with the highest AER to minimize the effect of efficient antihypertensive treatment. Finally, when multiple AER values were measured within one year, we used the geometric mean of the values to increase stability of the values.

While some overlap may exist between the genetic factors for chronic kidney disease in the general population, and the renal complications in patients with diabetes^26^, our observation that this association is only seen in individuals with high blood glucose levels supports the assumption that there are genetic factors specific for DN, and that these can only be identified in diabetic individuals. However, the genetics of DN remains poorly understood. DN is a heterogeneous complication affected by both glomerular filtration rate and urinary albumin excretion due to defects in the glomerular barrier and also exaggerated tubular reabsorption of glucose and sodium^27^. Since many of the previous genetic findings for DN were identified for the most severe form, ESRD^28^, our current observation represents the first locus with a genome-wide significant association and replication for albuminuria in diabetic individuals. Even though the association was only observed in the Finnish population, the finding may improve the biological understanding and profit the diabetic individuals worldwide once the functional mechanism behind the genetic association is revealed. Indeed, more functional work is required, since despite previous pilot sequencing, imputed GWAS data, and novel WES data, we cannot yet pinpoint the culprit causal variant or variants behind the observed association.

## Methods

### Patients

The present study included 3612 Finnish individuals with type 1 diabetes as diagnosed by their attending physician, age at diabetes onset no more than 40 years, insulin treatment initiated within two years of the diabetes diagnosis, and data on AER and genotypes available: N = 2864 patients with 24-hour (du-)AER [mg/24 hours], and N = 748 with overnight nu-AER [µg/min]. For patients with prevalent or incident ESRD, only values before ESRD were considered. The AER phenotype definition closely followed our previous publication on the topic^7^. AER values were log_10_ transformed. For individuals with multiple study visits, the visit with the highest AER value was selected in order to reduce the potential effect of efficient treatment. If multiple AER measures of the same type (nu-AER or 24-hour AER) were available within one year of the study visit, these were combined by calculating their geometric mean.

Whole exome sequencing (WES) data were available for 479 FinnDiane patients with type 1 diabetes, of whom 240 had rapid onset of macroalbuminuria (mean time from diabetes onset to macroalbuminuria 16 ± 3 years) or ESRD (20 ± 3 years; jointly, “cases”) and 239 with long duration of diabetes (43 ± 7 years) without diabetic nephropathy (“controls”)^29^. 24-hour AER measurements were available for 328 of these patients, of which 212 were classified as controls, and 116 (35%) as DN cases in the original analysis (Supplementary Fig. S4).

Patients gave their informed consent. The study was approved by the ethics committee of the Hospital District of Helsinki and Uusimaa and the local ethics committees, and the reported investigations were carried out in accordance with the principles of the Declaration of Helsinki as revised in 2008.

### Genotyping

A total of 6152 unique individuals with type 1 diabetes were genotyped on three batches with Illumina HumanCoreExome Bead arrays 12-1.0, 12-1.1, and 24-1.0 (Illumina, San Diego, CA, USA) at the University of Virginia. Variants were called with zCall^30^. After quality control to remove variants and subjects with low genotyping quality (e.g. minor allele frequency [MAF] < 0.01, genotyping rate <0.95), the variants were converted to human genome build 37 positive strand, and the batches were merged together. Genotype imputation was performed with minimac3-software^31^ using the 1000 Genomes phase 3 samples as the reference panel^12^. Within the 6019 samples passing the quality control, 3612 individuals had AER measurements and fulfilled the inclusion criteria. Imputation quality was good (r^2^ = 0.93) for the lead SNP rs10011025, and for the three other SNPs selected for replication in the original publication (r^2^ = 0.92–0.99). These four SNPs were also in Hardy-Weinberg equilibrium (*p* > 0.05). We further identified in the GWAS data 6690 non-monomorphic variants (i.e. MAC ≥1) with imputation quality r^2^ ≥0.8 and within 1 M base pairs (bp) of the SNP rs10011025, located on chromosome 4 at 175,654,223 bp.

Sequencing, quality control, variant calling and annotation of the WES data has previously been described^29^. Briefly, sequencing was performed at the University of Oxford on an Illumina HiSeq 2000 as part of a larger sequencing effort with other studies. An average 20-fold target capture was required for >80% of coverage. Mean sequencing depth was 54.97 for 497 included FinnDiane samples. Sequences were mapped with Burrows–Wheeler aligner v7.4^32^, and variants were called with Genome analysis toolkit (GATK) v2.1^33^. 188,068 polymorphic variants (MAC ≥1) remained for 479 unrelated FinnDiane individuals after quality control. Variants were annotated using CHAos (http://www.well.ox.ac.uk/~kgaulton/chaos.shtml), snpEff^34^ and VEP^35^ for functional class and transcript. Results from a meta-analysis of this and two other WES studies on DN have been previously reported^29^.

### Statistical analyses

Genetic association analysis was performed with the rvtests software (version 20160404)^36^ using the score test and adjusting for duration of diabetes, age at diabetes onset, use of antihypertensive medication, sex, and kinship matrix to account for related individuals and potential population stratification in the data set. Meta-analysis was performed with metal software (version 2011-03-25) based on effect size estimates^37^. While -log_10_ transformed AER values were used in replication to allow meta-analysis with previous findings based on the effect size estimates, inversed normal transformed residuals were used for analysis of the larger *GLRA3* region to optimize performance with rare variants. Variants were annotated with SNPnexus^38^.

For the WES data, single variant tests were performed in a similar way as for the GWAS analyses by using rvtests. To test enrichment of rare variants in genes, we used a kernel based gene aggregate method, SKAT-O, also implemented in rvtests^36^. SKAT-O and single marker tests were adjusted for sex and two principal components. AER was inverse normal transformed to optimize performance with rare variants. Relatives were excluded from the analyses.

## Electronic supplementary material

Supplementary material

## Data Availability

The datasets generated and/or analyzed during the current study are not publicly available as the patients’ written consent does not allow data sharing. Data are locally available from the corresponding author on reasonable request.
