# Supplementary material for: Confirmation of GLRA3 as a susceptibility locus for albuminuria in Finnish patients with type 1 diabetes
Source: Sci Rep. 2018 Aug 17;8:12408. doi: 10.1038/s41598-018-29211-1 (PMC6098108; doi:10.1038/s41598-018-29211-1)

**SUPPLEMENTARY MATERIAL**

**Confirmation of *GLRA3* as a susceptibility locus for albuminuria in Finnish patients with type 1 diabetes**

Niina Sandholm^1,2,3^, Jani Haukka^1,2,3^, Iiro Toppila^1,2,3^, Erkka Valo^1,2,3^, Valma Harjutsalo^1,2,3,4^, Carol Forsblom^1,2,3^, Per-Henrik Groop^1,2,3,5^ on behalf of the FinnDiane Study

1. Folkhälsan Institute of Genetics, Folkhälsan Research Center, FI-00290, Helsinki, Finland
2. Abdominal Center, Nephrology, University of Helsinki and Helsinki University Hospital, FI-00290, Helsinki, Finland
3. Research Programs Unit, Diabetes and Obesity, University of Helsinki, FI-00290, Helsinki, Finland
4. The Chronic Disease Prevention Unit, National Institute for Health and Welfare, FI-00271, Helsinki, Finland
5. Department of Diabetes, Central Clinical School, Monash University, Melbourne, Victoria, Australia

Contents

[**Supplementary Table S1: Missense variants within 1Mbp from rs10011025 (*GLRA3*) in GWAS data.** 2](#_Toc514155191)

[**Supplementary Table S2: Missense variants identified in *ADAM29* contributing to significant enrichment of missense variants associated with AER (SKAT-O *p*-value=0.009).** 3](#_Toc514155192)

[**Supplementary Table S3: Physicians and nurses at healt care centers participating in the collection of FinnDiane patients.** 4](#_Toc514155193)

[**Supplementary Figure S1: Effect size estimates for association between rs10011025 and 24-hour AER, stratified by the diabetes duration quartiles.** 7](#_Toc514155194)

[**Supplementary Figure S2: Proportion of SNPs within 1Mbp of rs10011025 with significant p-value (*p*<0.05/ <0.01/ <0.001), indicating enrichment of significant SNPs across all frequency categories.** 8](#_Toc514155195)

[**Supplementary Figure S3: Association between rs10011025 and AER within the 328 subjects with WES and 24-hour AER data available, after adjusting for uncommon (MAF<10%) missense variants in ADAM29.** 9](#_Toc514155196)

[**Supplementary Figure S4: Histogram of log_10_(24h AER) for FinnDiane subjects with WES data.** 10](#_Toc514155197)

# **Supplementary Table S1: Missense variants within 1Mbp from rs10011025 (*GLRA3*) in GWAS data.**

| **SNP** | **rs-number** | **Gene** | **MAF** | **MAC** | **MA Effect** | **SE** | **P** | **aa change** | **SIFT** | **Polyphen** |
| --- | --- | --- | --- | --- | --- | --- | --- | --- | --- | --- |
| chr4:175180938:T/C:1 | rs61748174 | *FBXO8* | 0.001 | 6.5 | -0.150 | 0.408 | 0.713 | N123S | TOLERATED | BENIGN |
| chr4:175229859:G/A:1 | rs147478051 | *CEP44* | 0.001 | 9.4 | 0.547 | 0.334 | 0.101 | R176H | **DAMAGING** | **PROBABLY DAMAGING** |
| chr4:175231175:C/G:1 | rs144338962 | *CEP44* | 0.000 | 2.3 | 1.123 | 0.813 | 0.167 | L285V | **DAMAGING** | **PROBABLY DAMAGING** |
| chr4:175564987:C/T:1 | rs144082170 | *GLRA3* | 0.018 | 132.2 | 0.060 | 0.090 | 0.505 | V449I | TOLERATED | BENIGN |
| chr4:175898555:C/T:1 | rs78280171 | *ADAM29* | 0.002 | 17.3 | 0.161 | 0.259 | 0.534 | P627S | TOLERATED | **POSSIBLY DAMAGING** |
| chr4:175898879:T/C:1 | rs113482601 | *ADAM29* | 0.001 | 10.8 | -0.193 | 0.328 | 0.557 | W735R | TOLERATED | BENIGN |
| chr4:175898934:A/G:1 | rs111721161 | *ADAM29* | 0.008 | 57.1 | 0.033 | 0.146 | 0.820 | Q753R | TOLERATED | UNKNOWN |
| chr4:175898967:C/T:1 | rs560490313 | *ADAM29* | 0.032 | 228.3 | -0.025 | 0.076 | 0.737 | T764I | TOLERATED | UNKNOWN |
| chr4:175899088:G/T:1 | rs146933346 | *ADAM29* | 0.016 | 118.5 | -0.061 | 0.110 | 0.581 | R804S | TOLERATED | UNKNOWN |
| chr4:175899091:A/T:1 | rs199709256 | *ADAM29* | 0.016 | 118.5 | -0.061 | 0.110 | 0.581 | Q805H | TOLERATED | UNKNOWN |

SNP: chromosome, base pair position, reference allele, alternative (minor) allele, and strand. MAF: minor allele frequency. MAC: minor allele count. MA Effect: β effect size estimate per one additional minor allele. SE: standard error for β. aa change: Affected amino acid and change introduced by the minor allele. SIFT: effect of the amino acid change on protein function as predicted by SIFT [1]. Polyphyen: effect of the amino acid change on protein function as predicted by polyphen [2].

**References:**

1. Kumar P, Henikoff S, Ng PC. Predicting the effects of coding non-synonymous variants on protein function using the SIFT algorithm. Nat Protoc 2009;4(7):1073-1081.

2. Adzhubei IA, Schmidt S, Peshkin L, Ramensky VE, Gerasimova A, Bork P, et al. A method and server for predicting damaging missense mutations. Nat Methods 2010;7(4):248-249.

# **Supplementary Table S2: Missense variants identified in *ADAM29* contributing to significant enrichment of missense variants associated with AER (SKAT-O *p*-value=0.009).**

| **POS** | **rs-number** | **REF** | **ALT** | **N** | **MAC** | **MAF** | **BETA** | **SE** | **P** | **aa change** | **description** | **SIFT** | **Polyphen** |
| --- | --- | --- | --- | --- | --- | --- | --- | --- | --- | --- | --- | --- | --- |
| chr4:175897719 |  | G | A | 328 | 1 | 0.002 | 1.062 | 1.004 | 0.288 | Arg348His | Non-synonymous | Tolerated (high) | Probably damaging |
| chr4:175898555 | rs78280171 | C | T | 328 | 3 | 0.005 | 0.864 | 0.581 | 0.136 | Pro627Ser | Non-synonymous | Tolerated (high) | Possibly damaging |
| chr4:175898743 | rs200852076 | TTTA | T | 328 | 56 | 0.085 | 0.341 | 0.141 | 0.015 | Leu691del | inframe deletion | NA | NA |

POS: chromosome and base pair position. REF: Reference allele. ALT: Alternative allele. N: number of individuals. MAC: minor allele count. MAF: minor allele frequency. BETA: β effect size estimate per one additional alternative (minor) allele. SE: standard error for β. P: p-value for single-variant association. aa change: Affected amino acid and change introduced by the minor allele. SIFT: effect of the amino acid change on protein function, (and confidence of prediction) as predicted by SIFT [1]. Polyphyen: effect of the amino acid change on protein function as predicted by polyphen [2].

**References:**

1. Kumar P, Henikoff S, Ng PC. Predicting the effects of coding non-synonymous variants on protein function using the SIFT algorithm. Nat Protoc 2009;4(7):1073-1081.

2. Adzhubei IA, Schmidt S, Peshkin L, Ramensky VE, Gerasimova A, Bork P, et al. A method and server for predicting damaging missense mutations. Nat Methods 2010;7(4):248-249.

# **Supplementary Table S3: Physicians and nurses at healt care centers participating in the collection of FinnDiane patients.**

| FinnDiane Study Centers | Physicians and nurses |
| --- | --- |
| Anjalankoski Health Centre | S. Koivula, T. Uggeldahl |
| Central Finland Central Hospital, Jyväskylä | T. Forslund, A. Halonen, A. Koistinen, P. Koskiaho, M. Laukkanen, J. Saltevo, M. Tiihonen |
| Central Hospital of Åland Islands, Mariehamn | M. Forsen, H. Granlund, A-C. Jonsson, B. Nyroos |
| Central Hospital of Kanta-Häme, Hämeenlinna | P. Kinnunen, A. Orvola, T. Salonen, A. Vähänen |
| Central Hospital of Länsi-Pohja, Kemi | H. Laukkanen, P. Nyländen, A. Sademies |
| Central Ostrabothnian Hospital District, Kokkola | S. Anderson, B. Asplund, U. Byskata, P. Liedes, M. Kuusela, T. Virkkala |
| City of Espoo Health Centre |  |
| Espoonlahti | A. Nikkola, E. Ritola |
| Tapiola | M. Niska, H. Saarinen |
| Samaria | E. Oukko-Ruponen, T. Virtanen |
| Viherlaakso | A. Lyytinen |
| City of Helsinki Health Centre |  |
| Puistola | H. Kari, T. Simonen |
| Suutarila | A. Kaprio, J. Kärkkäinen, B. Rantaeskola |
| Töölö | P. Kääriäinen, J. Haaga, A-L. Pietiläinen |
| City of Hyvinkää Health Centre | S. Klemetti, T. Nyandoto, E. Rontu, S. Satuli-Autere |
| City of Vantaa Health Centre |  |
| Korso | R. Toivonen, H. Virtanen |
| Länsimäki | R. Ahonen, M. Ivaska-Suomela, A. Jauhiainen |
| Martinlaakso | M. Laine, T. Pellonpää, R. Puranen |
| Myyrmäki | A. Airas, J. Laakso, K. Rautavaara |
| Rekola | M. Erola, E. Jatkola |
| Tikkurila | R. Lönnblad, A. Malm, J. Mäkelä, E. Rautamo |
| Heinola Health Centre | P. Hentunen, J. Lagerstam |
| Helsinki University Central Hospital, Department of Medicine, Division of Nephrology | A. Ahola, J. Fagerudd, M. Feodoroff, D. Gordin, O. Heikkilä, K Hietala, L. Kyllönen, J. Kytö, S. Lindh, K. Pettersson-Fernholm, M. Rosengård-Bärlund, M. Rönnback, A. Sandelin, A-R Salonen, L. Salovaara, L. Thorn, J. Tuomikangas, T. Vesisenaho, J. Wadén |
| Herttoniemi Hospital, Helsinki | V. Sipilä |
| Hospital of Lounais-Häme, Forssa | T. Kalliomäki, J. Koskelainen, R. Nikkanen, N. Savolainen, H. Sulonen, E. Valtonen |
| Iisalmi Hospital | E. Toivanen |
| Jokilaakso Hospital, Jämsä | A. Parta, I. Pirttiniemi |
| Jorvi Hospital, Helsinki University Central Hospital | S. Aranko, S. Ervasti, R. Kauppinen-Mäkelin, A. Kuusisto, T. Leppälä, K. Nikkilä, L. Pekkonen |
| Jyväskylä Health Centre, Kyllö | K. Nuorva, M. Tiihonen |
| Kainuu Central Hospital, Kajaani | S. Jokelainen, P. Kemppainen, A-M. Mankinen, M. Sankari |
| Kerava Health Centre | H. Stuckey, P. Suominen |
| Kirkkonummi Health Centre | A. Lappalainen, M. Liimatainen, J. Santaholma |
| Kivelä Hospital, Helsinki | A. Aimolahti, E. Huovinen |
| Koskela Hospital, Helsinki | V. Ilkka, M. Lehtimäki |
| Kotka Heath Centre | E. Pälikkö-Kontinen, A. Vanhanen |
| Kouvola Health Centre | E. Koskinen, T. Siitonen |
| Kuopio University Hospital | E. Huttunen, R. Ikäheimo, P. Karhapää, P. Kekäläinen, M. Laakso, T. Lakka, E. Lampainen, L. Moilanen, L. Niskanen, U. Tuovinen, I. Vauhkonen, E. Voutilainen |
| Kuusamo Health Centre | T. Kääriäinen, E. Isopoussu |
| Kuusankoski Hospital | E. Kilkki, I. Koskinen, L. Riihelä |
| Laakso Hospital, Helsinki | T. Meriläinen, P. Poukka, R. Savolainen, N. Uhlenius |
| Lahti City Hospital | A. Mäkelä, M. Tanner |
| Lapland Central Hospital, Rovaniemi | L. Hyvärinen, S. Severinkangas, T. Tulokas |
| Lappeenranta Health Centre | P. Linkola, I. Pulli |
| Lohja Hospital | T. Granlund, M. Saari, T. Salonen |
| Loimaa Health Centre | A. Mäkelä, P. Eloranta |
| Länsi-Uusimaa Hospital, Tammisaari | I-M. Jousmaa, J. Rinne |
| Malmi Hospital, Helsinki | H. Lanki, S. Moilanen, M. Tilly-Kiesi |
| Mikkeli Central Hospital | A. Gynther, R. Manninen, P. Nironen, M. Salminen, T. Vänttinen |
| Mänttä Regional Hospital | I. Pirttiniemi, A-M. Hänninen |
| North Karelian Hospital, Joensuu | U-M. Henttula, P. Kekäläinen, M. Pietarinen, A. Rissanen, M. Voutilainen |
| Nurmijärvi Health Centre | A. Burgos, K. Urtamo |
| Oulankangas Hospital, Oulainen | E. Jokelainen, P-L. Jylkkä, E. Kaarlela, J. Vuolaspuro |
| Oulu Health Centre | L. Hiltunen, R. Häkkinen, S. Keinänen-Kiukaanniemi |
| Oulu University Hospital | R. Ikäheimo |
| Päijät-Häme Central Hospital | H. Haapamäki, A. Helanterä, S. Hämäläinen, V. Ilvesmäki, H. Miettinen |
| Palokka Health Centre | P. Sopanen, L. Welling |
| Pieksämäki Hospital | V. Javtsenko, M. Tamminen |
| Pietarsaari Hospital | M-L. Holmbäck, B. Isomaa, L. Sarelin |
| Pori City Hospital | P. Ahonen, P. Merensalo, K. Sävelä |
| Porvoo Hospital | M. Kallio, B. Rask, S. Rämö |
| Raahe Hospital | A. Holma, M. Honkala, A. Tuomivaara, R. Vainionpää |
| Rauma Hospital | K. Laine, K. Saarinen, T. Salminen |
| Riihimäki Hospital | P. Aalto, E. Immonen, L. Juurinen |
| Salo Hospital | A. Alanko, J. Lapinleimu, P. Rautio, M. Virtanen |
| Satakunta Central Hospital, Pori | M. Asola, M. Juhola, P. Kunelius, M-L. Lahdenmäki, P. Pääkkönen, M. Rautavirta |
| Savonlinna Central Hospital | E. Korpi-Hyövälti, T. Latvala, E. Leijala |
| South Karelia Central Hospital, Lappeenranta | T. Ensala, E. Hussi, R. Härkönen, U. Nyholm, J. Toivanen |
| Tampere Health Centre | A. Vaden, P. Alarotu, E. Kujansuu, H. Kirkkopelto-Jokinen, M. Helin, S. Gummerus, L. Calonius, T. Niskanen, T. Kaitala, T. Vatanen |
| Tampere University Hospital | I. Ala-Houhala, T. Kuningas, P. Lampinen, M. Määttä, H. Oksala, T. Oksanen, K. Salonen, H. Tauriainen, S. Tulokas |
| Tiirismaa Health Centre, Hollola | T. Kivelä, L, Petlin, L. Savolainen |
| Turku Health Centre | I. Hämäläinen, H. Virtamo, M. Vähätalo |
| Turku University Central Hospital | K. Breitholz, R. Eskola, K. Metsärinne, U. Pietilä, P. Saarinen, R. Tuominen, S. Äyräpää |
| Vaajakoski Health Centre | K. Mäkinen, P. Sopanen |
| Valkeakoski Regional Hospital | S. Ojanen, E. Valtonen, H. Ylönen, M. Rautiainen, T. Immonen |
| Vammala Regional Hospital | I. Isomäki, R. Kroneld, M. Tapiolinna-Mäkelä |
| Vaasa Central Hospital | S. Bergkulla, U. Hautamäki, V-A. Myllyniemi, I. Rusk |

**Supplementary Figure S1: Effect size estimates for association between rs10011025 and 24-hour AER, stratified by the diabetes duration quartiles.** A total of 2864 subjects with updated GWAS data and 24-h AER were included in the analysis. Q1: duration 10 - 17.4 years; Q2 duration ≤ 24.8 years; Q3 duration ≤ 33.4 years; Q4 duration ≤ 65.8 years. Each quartile had 716 individuals.


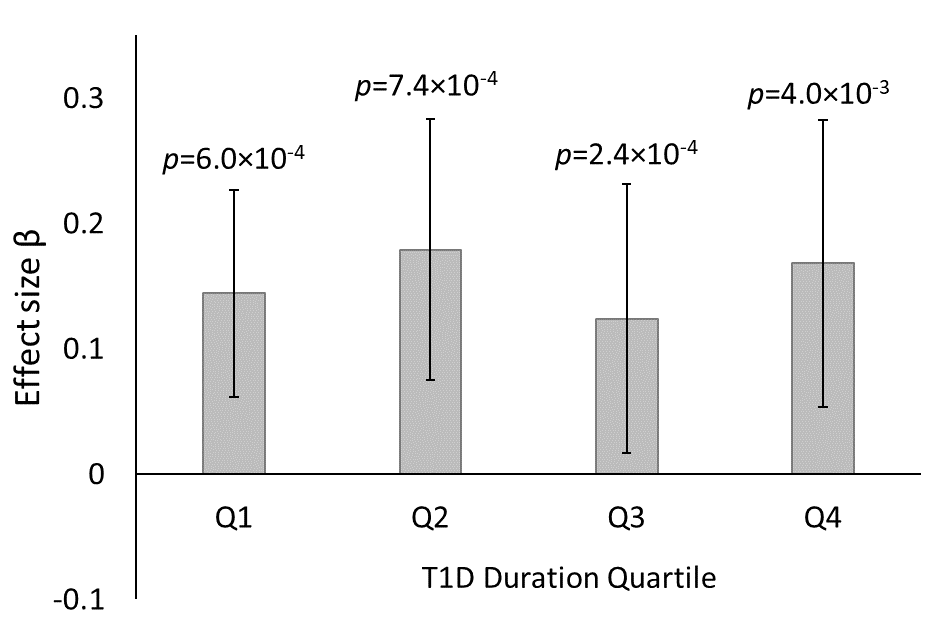


**Supplementary Figure S2: Proportion of SNPs within 1Mbp of rs10011025 with significant p-value (*p*<0.05/ <0.01/ <0.001), indicating enrichment of significant SNPs across all frequency categories.** Rare: minor allele frequency (MAF) <0.01; Low freq: 0.01 ≤ MAF < 0.05; Uncommon: 0.05 ≤ MAF < 0.1; Common: MAF ≥ 0.1. Background color indicates the expected proportion of SNPs with significant p-value under random association (e.g. 5% for p<0.05). Significant enrichment (one-tailed Bernoulli’s binomial test p<0.05) of significant p-values is indicated with an asterisk (*).


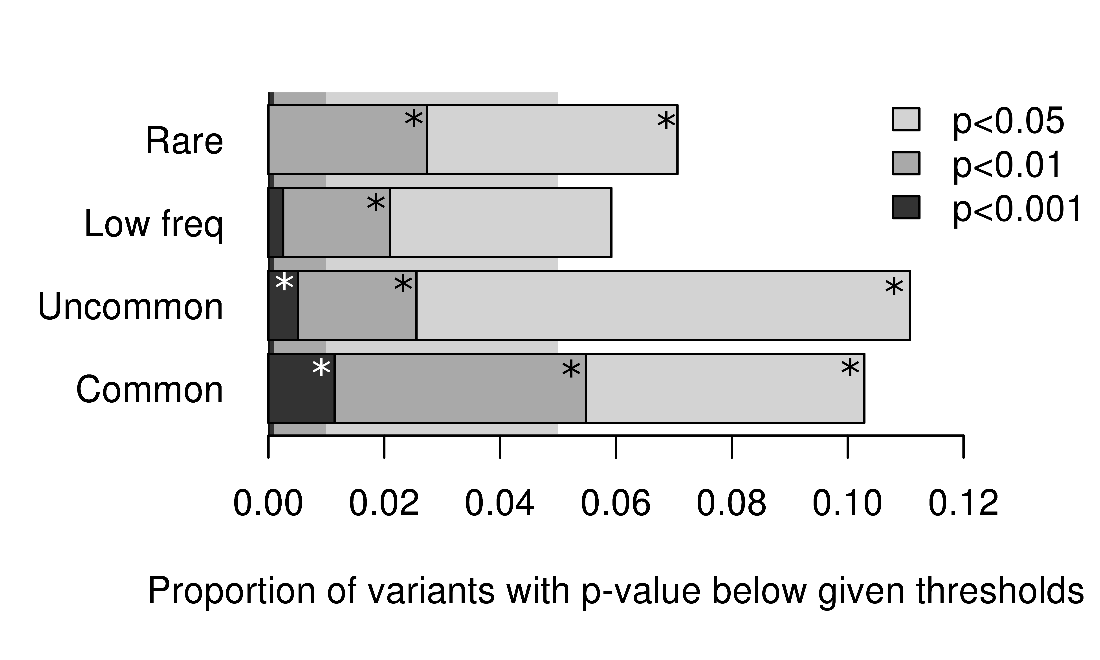


# **Supplementary Figure S3: Association between rs10011025 and AER within the 328 subjects with WES and 24-hour AER data available, after adjusting for uncommon (MAF<10%) missense variants in ADAM29.**


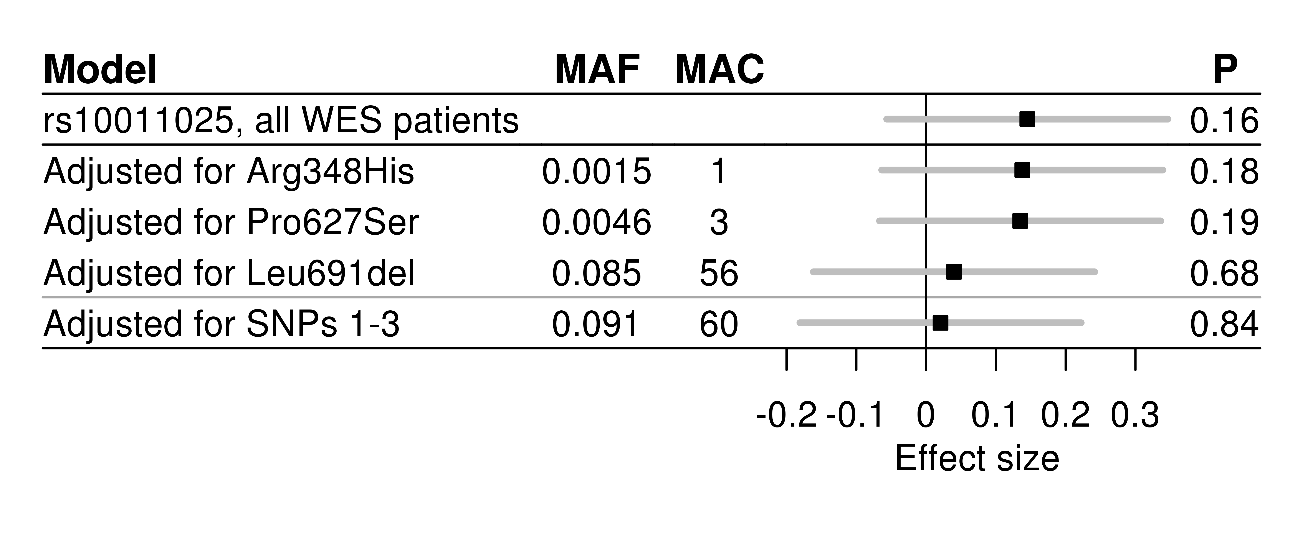


**Supplementary Figure S4: Histogram of log_10_(24h AER) for FinnDiane subjects with WES data.** Due to the non-normal distribution of the values, AER was inverse normal transformed for WES analyses.


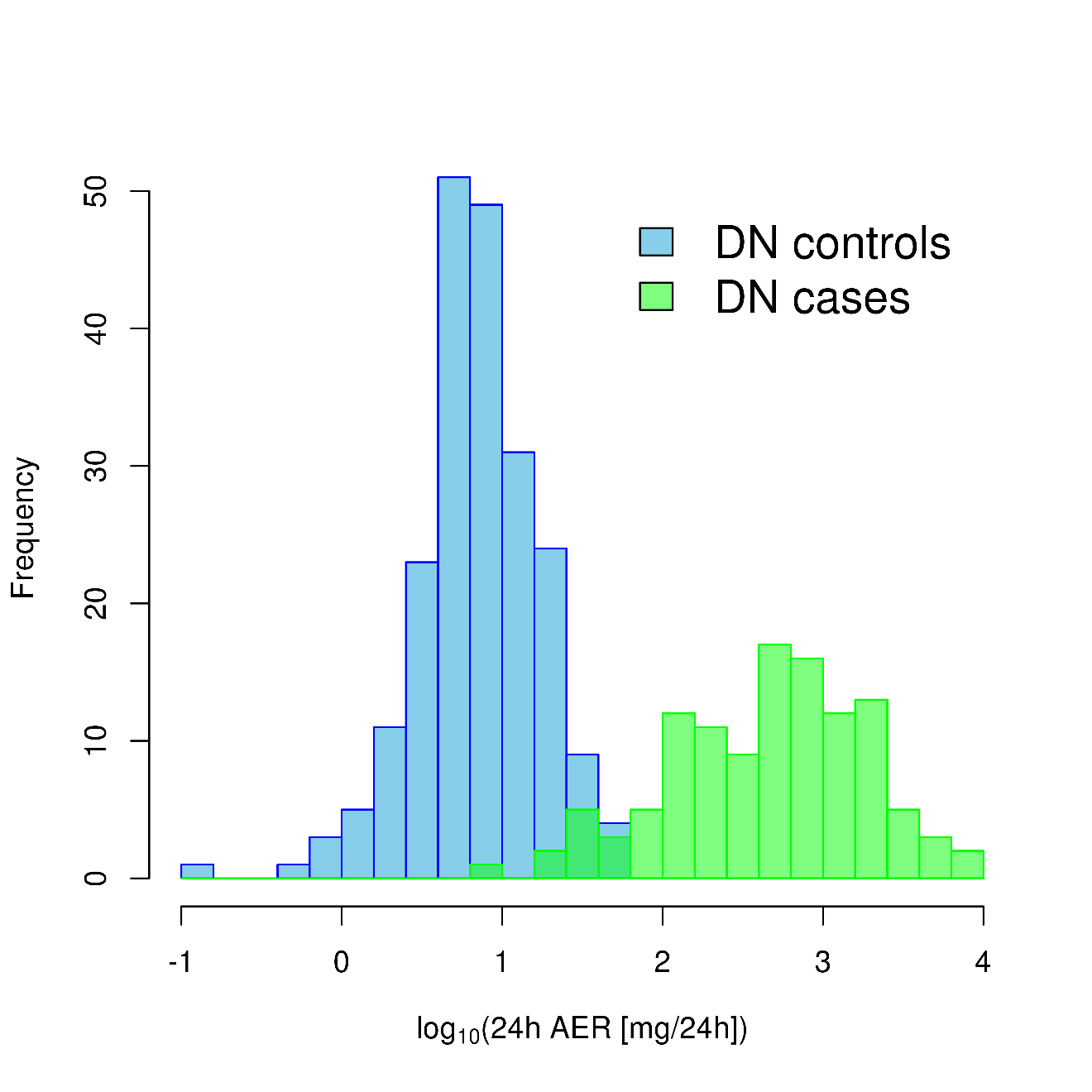

Supplement: Supplementary file 1 — Supplementary material [file 41598_2018_29211_MOESM1_ESM.docx]
